# Effectiveness of an intercultural module added to the treatment guidelines for Moroccan and Turkish patients with depressive and anxiety disorders

**DOI:** 10.1186/1471-244X-11-13

**Published:** 2011-01-19

**Authors:** Annelies van Loon, Digna JF van Schaik, Jack J Dekker, Aartjan TF Beekman

**Affiliations:** 1 Research Department, GGZ inGeest, Amsterdam, The Netherlands; 2 Department of Clinical Psychology, VU University Amsterdam, The Netherlands; 3 Research Department, Arkin Mental Health Institute, Amsterdam, The Netherlands; 4 Department of Psychiatry and Institute for Research in Extramural Medicine, VU University Medical Center, Amsterdam, The Netherlands

## Abstract

****Background**:**

Since the sixties of the last century, many people from Morocco and Turkey have migrated into the Netherlands. In the last decade, Moroccan and Turkish patients have found their way to organizations for mental health care. However, they often drop out of treatment. Problems in the communication with therapists and different expectations regarding treatment seem to be causal factors for the early drop-out of therapy. In the Netherlands as in other countries courses have been developed for training cultural competence of therapists. Yet, up to now, the effectiveness of increased cultural competence of therapists in reducing drop-out of treatment has not been studied.

****Methods/Design**:**

A randomized clinical trial was started in January 2010. Moroccan and Turkish adult patients who are referred to our outpatient clinics for mood and anxiety disorders are randomly assigned to mental health workers who are trained in a cultural module and to those who are not. The therapists have been trained in the Cultural Formulation and in techniques bridging the (cultural) gap between them and their Moroccan and Turkish patients.

The target number of participants is 150 patients, 75 for each group. Drop-out of treatment is the primary outcome measure. Secondary outcome measures are no-show and patients' perspective of care.

****Discussion**:**

The study will give an answer to the question whether increasing cultural competence of therapists reduces drop-out of treatment in Moroccan and Turkish outpatients with depressive and anxiety disorders.

**Trial Registration:**

The Dutch Cochrane Centre, NTR1989

## Background

Since the beginning of the 1960 s, large groups of male labour immigrants from Morocco and Turkey have come to the Netherlands. In the beginning they left their wives and children behind to be cared for by relatives, later they reunited with their families in the Netherlands. They settled down in the large cities such as Amsterdam, because relatively more unskilled labour jobs were available there. After the reunion with the families there was a decrease in immigration of this kind of immigrants around 1985. However, the migration from Morocco and Turkey unexpectedly rose again because children of the immigrants often married men or women from Morocco or Turkey. This contributed to a prolonged migration. As a result there is a first, a second and a third generation although it is hard to strictly separate these generations [1,2]. In 2010 about 349.000 Moroccans and 384.000 Turkish (first and second generation) reside in The Netherlands [3]. In Amsterdam 9.0% is of Moroccan and 5.2% of the population is of Turkish descent [4]. The Moroccan and Turkish population form the largest group of immigrants in the Netherlands as well as in Amsterdam.

It is known that migration can be a stressful process that can lead to mental illness [5,5]. Previous studies in Belgium and the Netherlands have found that common mental disorders are more prevalent among Moroccan and Turkish immigrants [6,7]. Other studies have shown that these groups have found their way to the mental health services [8,9] but treatment intensity seems to be less favourable [10] and high rates of treatment drop-out were found: 46%, among immigrant patients in specialized mental health service use compared to 24% among Dutch patients [11,12]. The high drop-out rates can lead to higher risks of chronicity of symptoms and prolonged disabilities.

The high drop-out rates among immigrants may be due to language problems, to different interpretations of symptoms and to different expectations of treatment [13]. Adequate treatment can only be given when a firm and steady working-relation can be established. It is thought that training therapists in cultural competences will bridge the gap between immigrant patients and their therapists. Without this competence health practitioners can easily fall prey to errors of diagnosis, inappropriate and poor treatment [14]. Training modules in intercultural competence courses have been developed for (mental) health workers. Therapists learn about the cultural background of specific immigrant groups. They learn to be aware of different notions of health and illness and they are trained in specific intercultural skills. Although widely applied there is, up to now, no evidence that training in cultural competence reduces drop-out of treatment or improves treatment outcome [15,16].

The aim of this study is to test whether a cultural competence training for therapists can reduce the treatment drop-out in Moroccan and Turkish patients with depressive and anxiety disorders in specialized mental health care. In order to test the effectiveness of this course we designed a randomized controlled trial. Our research questions are:

1. Does the intercultural competences training of therapists reduce treatment drop-out rates of Moroccan and Turkish patients with depressive and anxiety disorders in specialized mental health care?

2. Does the intercultural competences training of therapists reduce no-show rates, enhance the patient-therapist working alliance and patients' trust in care?

3. What are determinants of treatment dropout?

## Method/Design

### Study design

**Figure 1:**
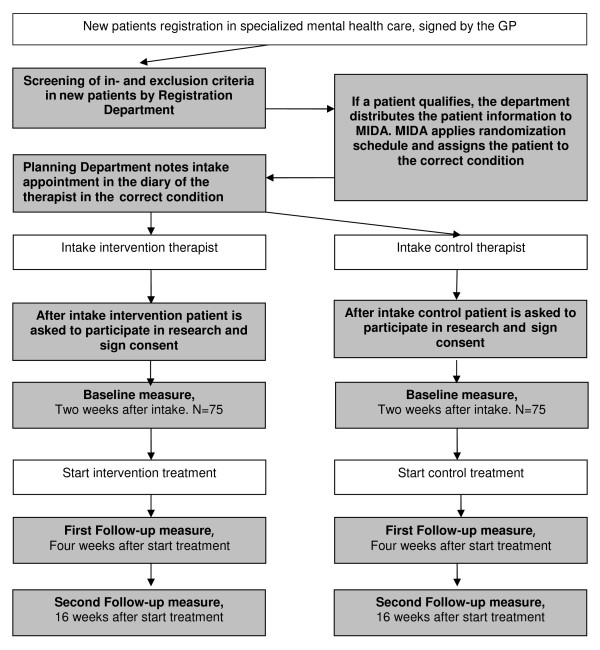
**Recruitment and measurement protocol**.

This study is a multi-centre randomized controlled trial in which treatment drop-out rates of Moroccan and Turkish immigrants with depressive and anxiety disorders, who are treated by therapists trained in cultural competences, are compared to those who receive regular care. Figure 1 shows the design.

### Recruitment/Setting and locations

Participants are recruited within two outpatient mood disorder clinics in Amsterdam. All new Moroccan and Turkish registries, mostly referred by their general practitioner, are screened on in- and exclusion criteria. If eligible, the new registries are randomized by a research assistant before the intake procedure starts. Then the patients are given an appointment for an intake interview by a therapist from the matching condition. After this intake interview the patients receive study information and the informed consent form from the therapists. Within two weeks after the intake an interviewer contacts a patient by telephone and asks oral consent to participate in the study. If the patient gives permission, an appointment is made for the basic interview in the clinic. If the patient is willing and eligible to participate, a written informed consent is signed.

### Randomization

The patients are randomized by an independent randomization team that uses a computerized random number generator. The patients are randomly assigned for either intake or treatment to therapists who are trained in cultural competence (the intervention group) or to therapists who are not trained in cultural competence (regular care, control group). The research assistant reveals the treatment condition by telephone to the registration staff who plans the intake interview.

### Participants

#### Inclusion criteria

Patients (ages 18 to 65) are eligible to participate if:

1. their main problem is a depressive and/or an anxiety disorder for which they are referred to the participating outpatient mood disorder clinics.

2. they are first or second generation Moroccan or Turkish immigrants. The definition for a first generation immigrant is that the patient him- or herself was born in Morocco or Turkey. The definition for the second generation migrant is that at least one of the patient's parents was born in Morocco or Turkey [17].

#### Exclusion criteria

Patients are excluded from the study if their main problem is one of the following disorders: a psychotic disorder, bipolar disorder, organic brain syndrome, substance dependence, or a severe borderline, schizotypical, or antisocial personality disorder.

### Intervention versus control-group therapists

In both clinics six therapists are selected for the intervention group and six for the control group of this study. In both groups disciplines of the therapists, the average amount of years of general treatment experience and ethnic background are evenly distributed.

### Intervention

Therapists of the intervention condition have been trained in cultural competence. The training program is based on existing modules that are widely used in the Netherlands and are based on international and national literature [18-23]. The aim of the module is to train the intervention therapists' knowledge, awareness and skills in diagnosing and treating Moroccan and Turkish patients with depression and anxiety disorders.

To improve the cultural knowledge the therapists are familiarized with the Moroccan and Turkish patients' background and learn about:

• the specific cultural aspect and elements,

• the impact of migration in first and second generation immigrants,

• the migration history,

• the religious background, (health)habits, beliefs, explanations and expectations,

• traditional health care and health workers.

To improve the cultural awareness therapists are taught to:

• be aware of their own cultural background, attitudes and values,

• recognise prejudices and generalizations regarding the cultural background of the patients.

To improve the intercultural skills the therapists are trained:

• how to give psychoeducation

• how to make use of an interpreter during treatment,

• how to make use of the Cultural Formulation [24] during intake and treatment,

• in intercultural diagnostics [25],

• how to use the intercultural addenda to the treatment guidelines for depression and anxiety disorders [26,27].

The module has an interactive character. It contains exercises in becoming aware of the therapists' own cultural background; a discussion about how to deal with traditional health workers; a role play with an actor in using an interpreter and in using the Cultural Formulation during treatment.

The training takes two days. After the training the intervention therapists join a monthly intercultural peer group to keep knowledge, awareness and skills vivid.

### Assessments

Data are collected at three points in time: within two weeks after the intake (T0), four weeks after the start of the treatment (T1), 16 weeks after the start of the treatment (T2). Table 1 summarizes the measures that are used at each point. The assessments are partly performed face to face by a trained interviewer and partly self report. Most of the interviewers are bilingual. Besides sufficient command of the Dutch language the Turkish, Moroccan-Arabic and Berber languages are requested respectively. The interviewers participate in a three day training. The interview and self report questionnaires consist of pen and paper versions only.

**Table 1:** Summary of measures

Primary outcome measure	Measurement instrument	Method	Baseline	Follow-up 1	Follow-up 2
Dropout	Medical Record	Report- therapist		X	X

					

**Secondary outcome measure**	**Measurement instrument**	**Method**	**Baseline**	**Follow-up 1**	**Follow-up 2**

***Course of treatment***					
Type of treatment	Medical Record	Report- therapist		X	X
Time in treatment	Medical Record	Report- therapist		X	X
No-show	Medical Record	Report- therapist		X	X
***Patients' perspective of treatment***					
Therapeutic relationship	HAQ	Self Report- patient	X	X	X
Patient evaluation of trust in care	NIVEL	Self Report- patient	X		X

					

**Determinants**	**Measurement instrument**	**Method**	**Baseline**	**Follow-up 1**	**Follow-up 2**

***Demographics***	Standard questions	Interview- patient			
			X		
***Public Health consequences***					
Level of functioning	WHO-DAS II	Self Report- patient	X	X	X
Work productivity	TIC-P	Interview- patient	X	X	X
Need of care	PNCQ Meadows	Interview- patient	X		X
***Anxiety and Depression***					
Diagnosis	CIDI	Interview- patient	X		
Severity of depression	IDS	Self Report- patient	X	X	X
Severity of anxiety	BAI	Self Report- patient	X	X	X
***Physical Conditions***					
Somatic disease	One- question	Interview- patient	X	X	X
Medication use	Medication use Questionnaire	Interview- patient	X		X
Pain	Chronic graded pain scale	Interview- patient	X		X
***Social functioning***					
Acculturation	LAS	Interview- patient	X		
Discrimination	Part of the National Survey of Midlife Development in US	Interview- patient	X		
Social functioning	Close Person Inventory	Self Report- patient	X		X

### Primary outcome measure

The primary outcome measure is drop-out of treatment. Treatment drop-out is defined as: the patient is in need of more therapy in the therapist's opinion but ignores at least two invitations of the therapist and does not continue the sessions. Data will be gathered by interviewing the therapists and analysing the medical records.

### Secondary outcome measures

Secondary outcome measures focus on no-show and the patients' perspective of treatment. No-show rates will be collected from the medical records of the participants. The patients' perspective will be measured using the Helping Alliance Questionnaire [28] and the trust in mental health care questionnaire (a part of the NIVEL consumer panel questionnaire will be used [29]).

### Dropout determinants

Several possible determinants of drop-out will be explored:

Demographic factors (age, gender, marital status, education); level of functioning (WHO-Disability Schedule II (WHO-DAS II) [30]); loss of productivity at work and health care utilization (Trimbos/iMTA questionnaire for costs associated with psychiatric illness (TIC-P) [31]); perceived need for care (Perceived Need for Care questionnaire (PNCQ) [32]); the diagnosis of depression and anxiety disorders (Composite International Diagnostic Interview (CIDI) depression and anxiety life time version (WHO version 2.1) ([33,33,35]); severity of depressive symptoms (Inventory of Depressive Symptoms self report version (IDS) [36]); severity of generalised anxiety and panic symptoms (Beck Anxiety Inventory self-report version (BAI)[37]); somatic disease (perceived somatic problems on an analogue scale from 1 to 10); medication use (Medication use questionnaire); pain (Chronic graded pain scale [38]); acculturation (the Lowlands Acculturation Scale (LAS) [39,40]); social support (Close Person Inventory [41]); discrimination (a part of the national survey of Midlife development in the US); locus of control (Pearlin and Schooler mastery scale [42]).

### Translation of the instruments

For this project insufficient command of the Dutch language is not an exclusion criterion. Therefore, we use translated questionnaires if necessary. For respondents who only understand the Turkish language, we use translated and validated Turkish instruments (IDS http://www.ids-qids.org, BAI [43], CIDI (WHO)) and translated instruments used in the Amsterdam Health Monitor (AHM) study [44] (Who-Das II, PNCQ, LAS, discrimination, locus of control, NIVEL trust in care). The other questionnaires were translated at our institute. Bilingual (Dutch and Turkish speaking and writing) mental health professionals translated the Dutch version into Turkish and this Turkish version was translated back into Dutch by other bilingual mental health professionals.

Because the Moroccan language is a collection of dialects no validated and translated versions of the instruments are available. For the respondents who only understand a Moroccan language we use several instruments used in the Amsterdam Health Monitor (AHM) (CIDI, LAS, discrimination, locus of control, PNCQ). These instruments were not completely translated into Moroccan Arabic. The Dutch version is used and only a keyword in each question was translated into Moroccan Arabic. For the other interview instruments (demographic, TIC-P, medication use, somatic disease, pain scale) keywords were translated in our institute by a focus group of bilingual students (Dutch-Moroccan-Arabic). For the self-report a complete translated version into Moroccan Arabic is accomplished. We asked the Dutch public translation centre (Tolk Vertaal centrum Nederland or TVcN) to translate the Dutch instruments into modern standard Moroccan Arabic. A focus group of bilingual students (Dutch-Moroccan-Arabic) translated it back into Dutch and adapted the Moroccan translation where necessary. We knew from the AHM study that most of the Moroccan respondents who did not speak Dutch well enough, speak a mix of Berber, Moroccan Arabic and Dutch. The interviewers are trained to cope with these language problems as much as possible. In all interviews the interviewers will specify in which language the interview has been conducted and how many problems they have encountered (language or verbal expression problems, associative behaviour or reluctancy to answer). Interviewers assess the reliability of the answers given.

### Sample size

No comparable intervention studies, aiming to reduce drop-out rates in immigrants, have been found in literature. In an intervention study by Blom et al [11,12], post hoc analyses showed that the drop-out rate was 46% in immigrant patients compared to 24% in native patients. Based on these data we assume that the drop-out can be reduced from 45% to 30%, an improvement of 33%. To demonstrate this difference 75 patients in each condition (beta 0.01 and alpha 0,05) should be included [45].

### Analysis

Basic characteristics will be compared between treatment conditions. Dropout will be analysed by (non) parametric longitudinal analysis techniques (eg GLM model) using multivariate statistics. Determinants are modelled along. Analysis will be performed according to the intention-to-treat principle.

### Ethical principles

Participation in this study is voluntary. Participants are informed that they can cancel their participation at any time without disclosing reasons for their cancellation and without negative consequences for their future care. Participants sign a written informed consent.

### Vote of the ethics committee

The design and conduct of the study was approved by the Medical Ethics Committee of the VU University Medical Center, Amsterdam.

## Discussion

This study protocol is presented here to offer researchers the opportunity to consider the methodological quality of this study with a critical view. Therapists can benefit by considering the information regarding the practical implications of the proposed protocol on immigrants with depressive and anxiety disorders in specialized mental health care.

The number of studies examining the effect of intercultural competence in mental health care is small [15,16]. High drop-out rates among immigrant patients is a serious mental health problem that deserves proper research. As far as we know, this is the first study examining the effect of intercultural competences for therapists on drop-out rates. Our study can make a contribution to the improvement of care for Moroccan and Turkish patients with depression and anxiety disorders in specialized mental health care in the Netherlands. Additionally findings may be generalised to other immigrants groups and to other countries.

This study is innovative in the development of a training module for specialised mental health therapists focusing on a specific treatment group, Moroccan and Turkish patients with depression and anxiety symptoms. We will systematically verify if the implementation of existing knowledge and skills are effective in improving working relationships and treatment. At the end of the study, we expect to define a clear and transferable intervention module, which, if effective, can be implemented in specialised mental health care.

### Positive aspect and limitations of the study

In this study we try to reach a study population, which is mostly excluded from clinical trials, as inadequate command of the dominant language is often an exclusion criterion. This is a positive aspect as well as a limitation of this study. Positive because findings can be generalised and are more representative for the whole group of immigrants; a limitation because most questionnaires are not validated.

Another positive aspect of this study is that due to the large overlap in instruments data from this project can be combined with those from the Amsterdam Health Monitor which contains mental health data from Amsterdam immigrants in first-line care [44]. In addition data from this study will be linked to those from the Dutch Study of Depression and Anxiety (NESDA), a large longitudinal study on depression and anxiety [46]. The number of Moroccan and Turkish participants enrolled in this study was small (N = 29). Therefore the findings of the present study provide valuable additional information. The questionnaires we use in the present study are largely consistent with the NESDA applied instruments, and combined data make it possible to explore psychometric properties of the translated instruments.

Other limitations of the study are that therapists will become aware of the intervention, and contamination between intervention and control group therapists can not be completely avoided. For a maximum prevention of contamination control therapists receive minimal information about the intervention. Intervention therapists discuss patients participating in the study in a separate supervision team on cultural competence.

### Description of risks

There are no specific risks related to this study.

## Competing interests

The authors declare that they have no competing interests.

## Authors' contributions

DJFS developed the design of the randomized clinical trial and participated in writing the article. JJD, and ATFB advised on the content of the article. AL is the principal investigator and writer of the manuscript. All authors have read and approved the final version of the manuscript.

## Pre-publication history

The pre-publication history for this paper can be accessed here:

http://www.biomedcentral.com/1471-244X/11/13/prepub
